# Study protocol for a pilot randomized controlled trial on a smartphone application-based intervention for subthreshold depression: Study protocol clinical trial (SPIRIT Compliant)

**DOI:** 10.1097/MD.0000000000018934

**Published:** 2020-01-24

**Authors:** Yudai Kato, Kaito Kageyama, Takanori Mesaki, Hiroyuki Uchida, Yoshiyuki Sejima, Risako Marume, Kana Takahashi, Kazuki Hirao

**Affiliations:** a Department of Occupational Therapy, Kibi International University, Takahashi; b Department of Rehabilitation, Kurashiki Heisei Hospital, Kurashiki; c Department of Rehabilitation, Okayama Kounan Hospital, Okayama, Japan.

**Keywords:** depression, eHealth, mHealth, mobile applications, smartphone, subthreshold depression

## Abstract

**Introduction:**

Interventions aimed at addressing subthreshold depression (StD) are important to prevent the onset of major depressive disorder. Our video playback application (SPSRS) is designed to reduce depressive symptoms by presenting positive words in videos, shedding new light on the treatment of StD. However, no randomized controlled trial (RCT) has utilized this video playback application for the treatment of individuals with StD. Therefore, a pilot RCT was designed to determine the feasibility of a full-scale trial. We herein present a study protocol for investigating the utility of a video playback application intervention for individuals with StD.

**Methods:**

This 5-week, single-blind, 2-arm, parallel-group, pilot RCT will determine the effectiveness of the video playback application by comparing individuals who had and had not been exposed to the same. A total of 32 individuals with StD will be randomly assigned to the experimental or control group at a 1:1 ratio. The experimental group will receive a 10-minute intervention containing the video playback application per day, whereas the control group will receive no intervention. The primary outcome will include changes in the Center for Epidemiologic Studies Depression Scale score after the 5-week intervention, while secondary outcomes will include changes in the Kessler Screening Scale for psychological distress and the generalized anxiety disorder 7-item scale score after the 5-week intervention. Statistical analysis using linear mixed models with the restricted maximum likelihood estimation method will then be performed.

**Discussion:**

This pilot RCT will have been the first to explore the utility of SPSRS application interventions that display positive words in videos for individuals with StD. The results of this pilot trial are expected to help in the design and implementation of a full-scale RCT that investigates the effects of SPSRS applications among individuals with StD.

**Trial registration:**

ClinicalTrials.gov Identifier: NCT04136041

## 1 Introduction

Subthreshold depression (StD) has been characterized as the presence of clinically significant depressive symptoms that do not satisfy the criteria for major depressive disorder (MDD).^[1]^ Previous studies have shown that StD has a prevalence rate of 4.0% to 38.7%,^[2–4]^ which is higher than that for MDD.^[5,6]^ Despite being a precursor to MDD,^[7]^ StD has been associated with poor quality of life,^[1,7,8]^ poor health,^[9]^ impaired daily activities,^[2,10]^ considerable economic burden,^[11]^ and mortality.^[12]^ Of particular importance, StD has been considered an significant risk factor for developing MDD given that many individuals with StD develop sustained depressive symptoms and are expected to progress to MDD.^[5,13,14]^ Moreover, MDD is likely to recur once established and often tends to be resistant to treatment.^[15]^ Therefore, intervention for StD is important to prevent the onset of MDD.

Previous studies have confirmed that several interventions are effective against StD.^[16–22]^ However, current reports have shown that medical costs for StD are nearing those for MDD.^[11]^ In addition, individuals with StD are believed to have poor motivation for intensive treatment.^[19]^ Considering the aforementioned limitations, alternative strategies that can easily relieve depressive symptoms among individuals with StD are needed. The SPSRS application we have developed, which is designed to improve depressive symptoms among individuals with StD by presenting positive words through video viewing, can address these issues and shed new light on StD treatment.^[23]^ SPSRS application interventions aim to relive depressive symptoms, thereby preventing progression to MDD and helping users manage their own health. Give that the SPSRS application uses the YouTube Application Programming Interface, users are able to select a favorite video and work on reducing depression symptoms while having fun. Moreover, considering that users need not change their daily routine (e.g., commuting to work), the SPSRS application creates an opportunity for addressing problems related to face-to-face treatment, which ultimately provides substantial benefits to the insurance system and society in general. Hence, we assume that SPSRS applications have great potential as new interventions for individuals with StD given that they reduce the psychological burden of an intervention. Although our previous study showed that SPSRS application interventions improved depressive symptoms among individuals with StD, it only compared outcomes before and after the intervention within a single group without establishing a control group.^[23]^ Therefore, randomized controlled trials (RCTs) based on rigorous methodology are essential to confirm the effectiveness of SPSRS applications for individuals with StD. However, to implement and achieve a high quality RCT, several methodological issues need to be considered and addressed (participant recruitment, administrative problems, harm, etc).^[24,25]^ In fact, reports have shown that several methodological issues have caused one-quarter of RCTs to be discontinued early.^[25]^ One of the best approaches for avoiding failure of a full-scale RCT has been to conduct a pilot RCT.^[26,27]^ Accordingly, a pilot RCT can determine the feasibility a full-scale RCT, increase the likelihood of its success, and prevent failure.^[26]^ Therefore, we herein outline our study protocol for a pilot RCT to investigate the utility of SPSRS application interventions for individuals with StD.

## 2 Objective

The present study aimed to determine the feasibility of a future full-scale RCT that will investigate whether SPSRS application interventions could improve depressive symptoms among individuals with StD relative to those receiving no intervention.

### 2.1 Trial design

This 5-week, single-blind, 2-arm, parallel-group, randomized, pilot RCT will determine the effectiveness of with the SPSRS application by comparing individuals who had and had not been exposed to the same.

## 3 Methods

### 3.1 Eligibility criteria

Participants will be selected according to the following eligibility criteria:

Inclusion criteria:

(1) Men and women
(2) 18 years and older
(3) Center for epidemiologic studies depression scale (CES-D) score ≥16.^[28]^
(4) Owns a smartphone with an iOS operating system.
(5) Written informed consent prior to participation.

Exclusion criteria:

(1) Lifetime history of psychiatric disorders.
(2) Currently receiving treatment for a mental health problem from a mental health professional.
(3) Vision or hearing deficits that negatively impact everyday life.
(4) Experience of a major depressive episode 2 weeks prior to the study ascertained using the Mini-International Neuropsychiatric Interview (MINI).^[29]^

### 3.2 Interventions

#### 3.2.1 Experimental group

The experimental group included herein will be scheduled to receive video viewing intervention using the SPSRS application designed for iPhones.^[23]^ The SPSRS application is a free video viewing application that uses the YouTube API, which allows individuals to search and watch videos using keywords, similar to a general video playback application. The SPSRS application is programmed to randomly display words that increase confidence, such as “can,” “let us try,” “good luck,” “able,” and “do not worry,” for 17 ms at the 4 corners of the screen.^[6]^ Thereafter, positive words, such as “nice,” “great,” “fantastic,” “satisfactory,” and “enjoyable,” are repeatedly displayed at the center of the screen for 150 ms every 5 seconds.^[30]^ The experimental group will receive a video viewing intervention using the SPSRS application for >10 minutes a day (>70 minutes per week). Given that the intervention period is scheduled for 5 weeks, the experimental group will be receiving > 350 minutes of video viewing intervention using the SPSRS application. Moreover, participants included herein will be able to freely select the videos to watch.

#### 3.2.2 Control group

The control group will receive no intervention and no instruction to watch videos using the SPSRS application or other video playback applications during the 5-week intervention period.

#### 3.2.3 Criteria for discontinuing or modifying allocated interventions

The intervention or follow-up is discontinued if any of the following occurs:

(1) Participant withdraws consent.
(2) The entire clinical trial is discontinued.
(3) Non-eligibility is confirmed after registration.
(4) The principal investigator or project administrator determines the need for discontinuation.
(5) A participant requires treatment for a mental health problem by a mental health professional following hospitalization or the onset of a mental disorder.

The date and reason for discontinuation will be documented in the case report. Participants will also be invited to participate in an outcome-related assessment to determine the effectiveness of the intervention. Participants who refuse to participate in follow-up measurements, provide data, or give consent will be considered withdrawn.

### 3.3 Strategies for monitoring and improving intervention protocol adherence

The amount of time participants spend on viewing videos during the intervention will be recorded in the history function of the SPSRS application and will be used for treatment adherence monitoring once a week. Participants who achieve the required video viewing duration per week (i.e., ≥70 minutes) will receive a text message to encourage continued video viewing. However, those who did not meet the required video viewing duration per week will receive a text message informing them regarding the same. In addition, a text message will be sent requesting the participant to make up for the missed viewing time on the subsequent week. Moreover, daily text messages will be sent to encourage the use of the SPSRS application and increase intervention adherence rates.

### 3.4 Relevant concomitant care and interventions permitted/prohibited during the trial

From an ethical point of view, no other intervention restrictions will be imposed in this study.

### 3.5 Outcomes

The primary outcome will include changes in the CES-D score^[28]^ after the 5-week intervention, while the secondary outcome will include changes in the Kessler Screening Scale for psychological distress (K6)^[31]^ and the generalized anxiety disorder 7-item scale (GAD-7)^[32]^ score following the 5-week intervention.

### 3.6 Participant timeline

**Figure 1:**
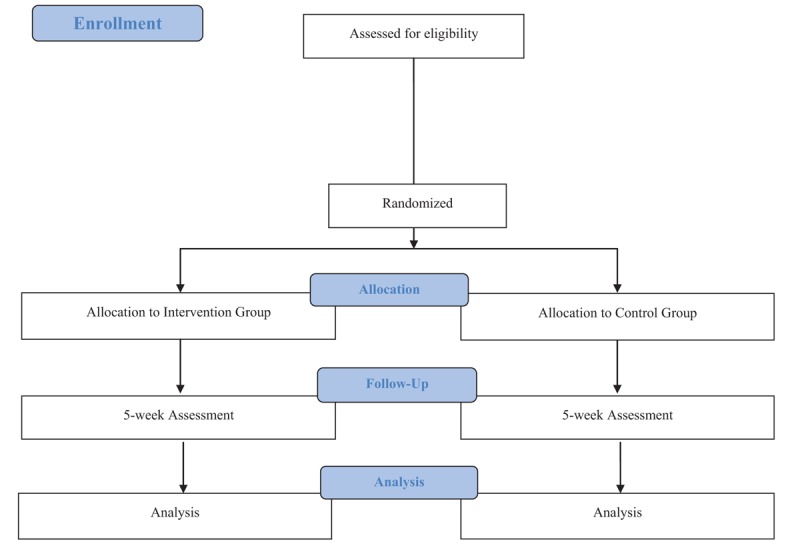
CONSORT flowchart of the study design.

Individuals interested in participating in this study will be assessed for eligibility using interviews and Internet questionnaires. Eligible participants will then be enrolled in the study and randomly assigned to the intervention or control group. The trial period is shown in the consolidated standards of reporting trials diagram^[33]^ (Fig. 1) and Standard Protocol Items: Recommendations for Interventional Trials template^[34]^ (Table 1).

**Table 1:**
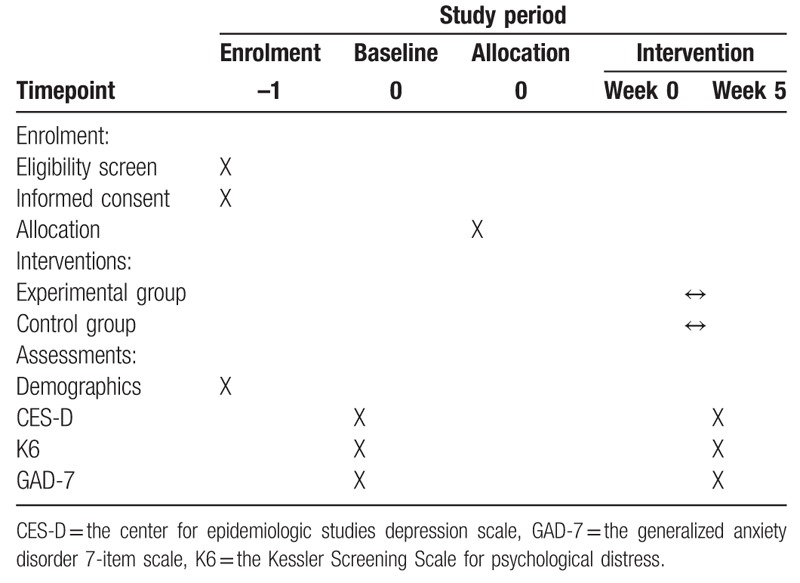
Assessment schedule.

### 3.7 Sample size

Four factors are required to calculate the sample size: significance level, power, difference between groups, and standard deviation (SD).^[35]^ However, considering that no previous 2-arm trial has used the SPSRS application for individuals with StD, values for the difference between groups and SD remain unknown. Although a pilot RCT does not require sample size calculations using the aforementioned 4 factors,^[26]^ 15 to 20 participants per group are required to ensure scientific validity of the pilot study results.^[36]^ Therefore, the present study will include a total of 32 participants with 16 participants per group. In addition, the results of this pilot study will provide information on the difference between groups and SD necessary for sample size calculation during the future full-scale study.

### 3.8 Study setting and recruitment

Potential participants will be recruited from October 2019 to March 2021 mainly in Okayama Prefecture, Japan through Kibi International University. The research will be promoted widely throughout the Kibi International University Takahashi Campus using advertising flyers. Many facilities will be made available for use by individuals other than students at the Kibi International University Takahashi Campus, while a considerable number participant will be expected given the university's close ties with local communities and surrounding organizations. In addition, the researchers will promote participation using emails and social networking service. All distributed materials will include a dedicated email address through which the research team may be contacted for further information on the study.

### 3.9 Allocation

Participants will be required to fill out eligibility and baseline questionnaires accessible through the Internet in addition to completing an interview. Only qualified individuals who provided consent will be randomly assigned to the experimental or control group. Participants will then be informed regarding the allocation results to increase study transparency. The random assignment will be performed by the Central Registration Center located at Kurashiki Heisei Hospital, Okayama Prefecture. The randomized list will be generated using an Excel spreadsheet by a third party not involved in this study and will be provided to the Central Registration Center. The randomized list will be created using a reasonably sized permuted block method, the block size of which will not be disclosed until the end of this trial to ensure concealment.

### 3.10 Blinding

Considering the unique nature of the intervention, participants and therapists cannot be blinded. Therefore, this trial will employ an Internet questionnaire to blind the assessors.

### 3.11 Data collection

Before the intervention begins, participants in the experimental group will be provided with a standardized SPSRS application manual and will be trained on how to operate the SPSRS application. To ensure data accuracy and reliability, researchers will undergo training sessions on data collection and storage. In addition, regular meetings will be held to address issues related to trial implementation. Outcome evaluation will follow the schedule outlined in Table 1. Demographic data, such as age, sex, sleep time, smoking habits, exercise habits, and drinking habits, will then be collected as part of the baseline assessment.

#### 3.11.1 Mini-international neuropsychiatric interview

The mini-international neuropsychiatric interview (MINI) is structured based on Diagnostic and Statistical Manual of Mental Disorders, 4th Edition and International statistical classification of diseases and related health problems, 10th revision.^[29]^ The present study will be using the Japanese version of the latest MINI (5.0.0), which contains 16 modules for psychiatric diagnosis (major depressive episodes, dysthymia, suicide risk, manic and hypomanic episodes, panic disorders, agoraphobia, social phobia, obsessive–compulsive disorder, posttraumatic stress disorder, alcohol use disorder, substance use disorder, psychotic disorder, anorexia nervosa, bulimia nervosa, generalized anxiety disorder, and antisocial personality disorder), to which respondents may answer with only “Yes” or “No.” The reliability and validity of the Japanese version of the MINI have already been confirmed.^[29]^

#### 3.11.2 Center for epidemiologic studies depression scale

The CES-D is a 20-item self-reported questionnaire that measures depressive symptoms using 4 subscales related to “somatic and retarded activity,” “depressed affect,” “positive affect,” and “interpersonal problems.”^[28]^ The CES-D uses a 4-point Likert scale (A = <1 day, B = 1–2 days, C = 3–4 days, and D = 5–7 days), with each item being scored 0 to 3, respectively. Participants may have a total score of 0 to 60 points, with a higher score indicating stronger depressive symptoms. The reliability and validity of CES-D have already been verified.^[28,37,38]^

#### 3.11.3 Kessler screening scale for psychological distress

The K6 is a 6-item self-report questionnaire that measures psychological distress using 4 questions about depression and 2 about anxiety.^[31]^ The K6 uses a 5-point Likert scale (4 = all of the time, 3 = most of the time, 2 = some of the time, 1 = a little of the time, and 0 = none of the time). Participants may have a total score of 0 to 24 points, with a higher score indicating stronger psychological distress. The reliability and validity of K6 have already been verified.^[31,39–43]^

#### 3.11.4 The generalized anxiety disorder 7-item scale

The GAD-7 is a 7-item self-report questionnaire that measures generalized anxiety disorder using a 4-point Likert scale (0 = not at all sure, 1 = several days, 2 = over half the days, 3 = nearly every day).^[32]^ Participants may have a total score of 0 to 21 points with a higher score indicating stronger anxiety symptoms. The reliability and validity of GAD-7 have already been verified.^[32,44–47]^

### 3.12 Promoting participant retention and completion of follow-up

Participants will be informed that the use of the SPSRS application and outcome measurements will be free of cost and that the SPSRS application will allow them to freely select videos to watch so that they can enjoy the intervention. In addition, participants will be provided a dedicated email address through which they may raises their concerns regarding the use of the SPSRS application. Moreover and if necessary, participants in the control group can receive video viewing interventions using the same SPSRS application after measuring outcomes 5 weeks following the start of the intervention. Outcome measurements will be conducted through an easy-to-use Internet questionnaire for the participant's convenience. Participants who satisfy the criteria for refusal or discontinuation of intervention will be asked to participate and provide data for the week 5 assessment. The aforementioned requests and responses will be explained to participants upon obtaining informed consent. No financial or physical incentives will be provided to participants at any point during the evaluation or intervention.

### 3.13 Data management

Data will be managed in accordance with methods used in previous studies.^[48–50]^ Accordingly, first and second stage management will be implemented to ensure data accuracy. First stage management will require 2 independent researchers to enter data into an Excel spreadsheet using a computer not connected to the Internet. The input data will then be saved onto a password-protected Universal Serial Bus. Second stage management will require 2 different researchers not involved in the first stage to confirm and double-checked for any potential errors in the input contents. Only individuals authorized by the principal investigator will be allowed access to the database. The Internet questionnaire data of each participant will be printed on paper and deleted after data entry. Paper materials related to each participant's evaluation will be stored in an evaluation binder, while intervention materials will be stored in a separate intervention binder. Binders that store evaluation and intervention forms will be kept inside a lockable shelf at Kibi International University. Paper files will be stored for up to 5 years after the examination ends.

### 3.14 Statistical methods

Intention-to-treat analysis will be employed herein using data obtained from all assigned participants. A linear mixed models with the restricted maximum likelihood estimation method will then be used to address values missing from the clinical trial, estimate the mean of the primary and secondary outcomes, and compare the experimental and control groups.^[51]^ Group assignment, evaluation time, and interactions between group assignment and evaluation time will be considered fixed effect factors, while participants will be considered random effect factors. The model will use a fixed effect type III test. A 2-sided *P* value of <.05 will be used to indicate statistical significance. No plans for subgroup analysis are currently in place. The latest version of SPSS (SPSS Japan Inc., Tokyo, Japan) will be used for data analysis. In addition, the effect size (Hedge *g*) between the 2 groups and its 95% confidence interval will also be reported.^[52,53]^

### 3.15 Data monitoring

This pilot RCT has been designed for conciseness and minimizing risk. Therefore, no formal data monitoring committee has been organized. In addition, no interim analysis of the intervention's impact has been planned at this stage.

### 3.16 Harm

Previous studies have reported no adverse events with the use of the SPSRS application. However, adverse events will be systematically monitored based on the methods used in previous studies,^[54]^ while prompt responses will be provided for incidences and signs of adverse events. Participants are encouraged to identify signs of mental or physical health deterioration upon study enrollment, regardless of whether they participate the intervention. The appearance of such symptoms will be considered an adverse event.

Participants will be taught 2 methods of identifying adverse events during and after the study. First, participants will be encouraged to talk to their researchers upon noticing signs of mental or physical health deterioration. Second, all study participants will be requested to inform the research team whether and why they plan to discontinue with the study. Upon the occurrence of an adverse event, the participant will be contacted and appropriate action will be taken. The investigator will also evaluate and document the participant's relationship with the study in addition to the content, duration, severity, and outcome of the adverse event.

Details regarding adverse events will be immediately reported to the Kibi International University Ethics Review Board who will then determine the direction of the study.

### 3.17 Auditing

No audit has been planned at this time.

### 3.18 Research ethics approval, protocol amendments, and consent

This trial has been approved by the Ethics Review Committee of Kibi International University (approval number: 19–33). Prior to participation, the principal investigator or collaborators will inform the participants regarding the details of this trial using trial information materials. Changes to the study protocol will require approval from the Kibi International University Ethics Review Board. Before obtaining informed consent, the researchers will briefly explain the potential risks and benefits of study participation. Documentation of the participants’ satisfaction with the risks and benefits is essential. The researchers will also inform participants that participation is optional and can be discontinued at any time. Informed consent, which will be presented through an Internet questionnaire and obtained by pressing the consent button when the participant agrees to participate in the trial, will be obtained before any intervention is provided.

### 3.19 Confidentiality

All information related to the study will be stored securely at Kibi International University. Paper documents will be stored separately for each participant. All survey materials will be identified using participant numbers and stored inside a lockable shelf to maintain participant confidentiality. Information that could potentially identify a participant, such as informed consent, will be kept separate from the survey material identified using participant numbers. The electronic database will be stored on a password-protected Universal Serial Bus and stored together with the paper survey materials. Information regarding a participant's identity will not be entered into the electronic database. Data input and statistical analysis will be conducted on a computer not connected to the Internet at Kibi International University.

### 3.20 Access to data

Prior to the publication of major results, only data managers and principal investigators will have access to the complete dataset. Data managers and principal investigators will address problems related to the data and finalize a dataset for statistical analysis. After publication, only the principal investigator and individuals approved by the principal investigator will have access to the dataset.

### 3.21 Ancillary and post-trial care

Participants will be able to contact the researchers during the trial period or until 1 month after the end of participation. This trial cannot guarantee the absence of unexpected serious adverse events during and after the participation period. Participants who develop serious adverse events will be responded to promptly and appropriately even after study completion.

### 3.22 Dissemination policy

Regardless of the outcome, the trial results will be published in a peer-reviewed journal. Furthermore, participating in and presentations at relevant national and international academic conventions can further promote dissemination.

## 4 Discussion

StD has been considered an important risk factor for the development of MDD.^[5,13,14]^ Moreover, despite being a precursor to MDD,^[7]^ StD has been associated with poor quality of life,^[1,7,8]^ poor health,^[9]^ impaired daily activities,^[2,10]^ economic costs,^[11]^ mortality,^[12]^ etc. Therefore, interventions aimed at addressing StD are important to prevent the onset of MDD. Incorporating the SPSRS application into interventions is a very innovative method that places less burden on individuals with StD and may improve depressive symptoms. In addition, given the potential for the SPSRS application to influence numerous individuals with StD, it may play an important role in addressing depressive symptoms that may have a significant negative impact on their health. However, RCTs using the SPSRS application for individuals with StD have been lacking. Therefore, a pilot RCT investigating the feasibility of a large-scale trial to determine the effectiveness of the SPSRS application had been planned. The results of this pilot RCT will undoubtedly ensure the successful implementation of a full-scale RCT. In addition, this pilot trial will provide new information through which a higher-quality full-scale RCT can be planned. A particularly important additional information obtained from results of this pilot RCT is that related to the official sample size calculation. Thus, this pilot RCT can provide evidence that reinforces the standard practice of SPSRS application interventions to improve depressive symptoms among individuals with StD.

This study, however, is not without limitations. First, this pilot study will utilize simple randomization. As a result, other demographic factors, such as age, can lead to sample heterogeneity. Future studies will need to consider stratification of various demographic factors. Second, given the restrictions on the use of the SPSRS application, participants must own an iPhone, thereby excluding those using smartphones running on other operating systems (such as Android). Therefore, our research results may not be generalized to all individuals with StD people. However, we plan to make the SPSRS application compatible with other smartphones (such as Android) for future research, which would considerably increase the number of potential participants. Third, a formal sample size calculation could not be performed given that lack of data on the use of the SPSRS application. This lack of sample size calculation may decrease the power of this pilot RCT. However, retrospective sample size calculation will be performed after this trial. Finally, given the nature of this study, participant and therapist blinding will be difficult. Therefore, some therapist and participant biases may be present. However, we will endeavor to mitigate potential biases resulting from the lack of blinding. For example, research hypotheses will be concealed and standardized intervention guidance and textual feedback will be provided to maintain data quality. Considering the aforementioned limitations, care should be taken in the interpretation of this pilot study's results.

## 5 Conclusion

This pilot RCT will have been the first to explore the utility of SPSRS application interventions, which display positive words in videos, for individuals with StD. The results of this pilot RCT are expected to help in the design and implementation of a full-scale RCT investigating the effects of the SPSRS application among individuals with StD.

## Author contributions

**Conceptualization:** Yudai Kato, Kaito Kageyama, Takanori Mesaki, Hiroyuki Uchida, Kazuki Hirao.

**Data curation:** Yudai Kato, Kaito Kageyama, Takanori Mesaki.

**Formal analysis:** Hiroyuki Uchida, Kazuki Hirao.

**Funding acquisition:** Kazuki Hirao.

**Investigation:** Yudai Kato, Kaito Kageyama, Takanori Mesaki, Yoshiyuki Sejima, Risako Marume, Kana Takahashi.

**Methodology:** Hiroyuki Uchida, Kazuki Hirao.

**Project administration:** Kazuki Hirao.

**Supervision:** Kazuki Hirao.

**Writing – original draft:** Hiroyuki Uchida, Kazuki Hirao.

**Writing – review & editing:** Yudai Kato, Kaito Kageyama, Takanori Mesaki, Hiroyuki Uchida, Yoshiyuki Sejima, Risako Marume, Kana Takahashi, Kazuki Hirao.

Kazuki Hirao orcid: 0000-0002-2467-7564.
